# The Influence of Mineral Wool on the Properties of the Mineral–Asphalt Mixture

**DOI:** 10.3390/ma19122475

**Published:** 2026-06-09

**Authors:** Magdalena Krzemińska, Grzegorz Rogojsz, Tomasz Rudnicki

**Affiliations:** 1 2nd Masovian Engineering Regiment, 4C Leśna Str., 05-100 Nowy Dwór Mazowiecki, Poland; magdalena.pak1434@gmail.com; 2 Faculty of Civil Engineering and Geodesy, Military University of Technology, Gen. Sylwestra Kaliskiego 2 Str., 00-908 Warsaw, Poland; tomasz.rudnicki@wat.edu.pl

**Keywords:** mineral wool, fiber-reinforced asphalt concrete

## Abstract

In this article, the authors presented the results of research evaluating the impact of mineral wool on the properties of asphalt mixtures intended for the wearing course. The primary goal of the research was to assess the suitability of granulated mineral wool as a modifier for asphalt mixtures, improving their strength and performance parameters. The research methodology was selected in accordance with the requirements of national technical documents. The analysis of the mineral wool’s impact was conducted for AS 11 S asphalt concrete at 0.5%, 1.0%, and 1.2% by weight of the asphalt mixture. Based on the research, it was concluded that the use of mineral wool reduced the stability of the samples and increased settlement in the Marshall test, which was its only negative effect. Furthermore, the addition of 1.0% mineral wool increases the resistance of samples to water and frost by 31%, while the addition of 1.2% mineral wool increases the stiffness of asphalt concrete by 35% at a reference temperature of 5 °C and by 15% at reference temperatures of 10 °C and 15 °C. It was found that the use of mineral wool as an additive allows for a reduction in the maximum rut by up to 50% and the slope of the rutting graph by 73%.

## 1. Introduction

In Poland, asphalt pavements dominate the national road network, accounting for approximately 98% of asphalt pavements, while concrete pavements represent only about 2% of the network. On motorways, asphalt is used on approximately 79% of the length, and on expressways on approximately 89%, confirming the dominant role of asphalt pavements in the national road network [1]. Asphalt pavements play a key role in road infrastructure, ensuring safety, driving comfort, and the durability of the entire transport system.

During service, pavements gradually deteriorate due to both environmental factors and mechanical loads generated by vehicle traffic [2,3,4,5,6,7]. The aging process of mineral–asphalt mixture involves physicochemical changes in its structure, such as oxidation, loss of light fractions, and an increase in stiffness and brittleness. These phenomena reduce the binder’s ability to relax stresses, which increases the pavement’s susceptibility to cracking, especially at low temperatures [6,8,9]. In addition, UV radiation and moisture accelerate degradation processes, weakening the adhesion between the binder and the aggregate and promoting structural damage in the mixture.

In parallel with the loss of chemical properties, mechanical properties also deteriorate under repeated axle loads. Repeated stresses lead to the initiation and propagation of microcracks, which eventually coalesce into fatigue cracks visible on the pavement surface. This phenomenon is particularly pronounced on roads carrying heavy traffic, where significant tensile stresses occur in the lower layers of the pavement structure [10]. As a result, load-bearing capacity gradually decreases, susceptibility to permanent deformation increases, and rutting develops, all of which significantly shorten pavement service life and generate high maintenance costs.

In response, intensive research has been conducted in recent years on the modification of MMA through the addition of various additives intended to improve its properties [11,12]. The literature pays particular attention to modifications aimed at increasing resistance to permanent deformation, fatigue cracking, and variable weather conditions, which strongly influence the rate of deterioration of road structures [13,14].

One of the most frequently studied groups of additives includes mineral additives, such as active fillers and fine mineral fractions, which improve the internal structure of MMA and enhance binder adhesion to aggregate. Research indicates that their use increases mix stiffness, improves water resistance, and reduces susceptibility to fatigue damage and permanent deformation [5,15]. It is particularly important to limit binder stripping from aggregate, which is one of the main causes of pavement deterioration under cyclic moisture and freezing conditions.

Another important group of modifiers includes polymers, such as SBS, elastomers, and epoxy resins, which significantly affect the rheological properties of asphalt binder. The use of polymers improves mixture flexibility at low temperatures and enhances resistance to rutting at elevated temperatures [16,17]. The literature emphasizes that polymer-modified asphalt mixtures are characterized by slower aging, greater resistance to thermal cracking, and better fatigue performance compared with unmodified mixtures. This leads to a longer service life and reduced maintenance frequency.

Fibers used as dispersed reinforcement in mineral–asphalt mixtures (MMAs), including glass, basalt, polymer, and recycled fibers, have become one of the most extensively investigated groups of modifiers in recent years [18,19,20,21]. Their growing popularity results both from the need to improve pavement durability under increasing traffic loads and from efforts to reduce the environmental impact of road construction through the use of alternative and recycled materials. Fibers improve stress distribution within the mixture and limit the initiation and propagation of microcracks, thereby enhancing fatigue resistance and extending pavement service life [15,22,23,24]. Moreover, fiber reinforcement contributes to improved resistance to moisture- and frost-induced damage by stabilizing the internal structure of the mixture and strengthening the adhesion between asphalt binder and aggregate. Numerous studies have demonstrated that fibers can significantly reduce rutting susceptibility and improve the overall mechanical performance of asphalt mixtures under heavy traffic conditions. The beneficial effect of fibers is commonly attributed to the formation of a three-dimensional reinforcing network within the asphalt mastic. This network improves stress transfer, restricts aggregate movement, limits crack propagation, and enhances the cohesion of the asphalt matrix [25]. Consequently, fiber-modified mixtures generally exhibit improved resistance to permanent deformation, fatigue damage, and environmental deterioration compared with conventional mixtures.

Particularly favorable results were obtained for glass, basalt, and polymer fibers. The authors of [20] demonstrated that the addition of glass wool fibers increases the indirect tensile strength (ITS) of the asphalt concrete mix, improves water resistance, and reduces the susceptibility of the mixes to rutting. Similar conclusions were presented in [26], where the authors found that glass fibers improve the volumetric properties of the mixes, and increase resistance to moisture damage and permanent deformation, with the effectiveness of the modification depending on both the content and length of the fibers used. In [27], the authors demonstrated the beneficial effect of basalt fibers on the rheological properties of asphalt mastics, while in [24] the authors confirmed that basalt, polyester and lignin fibers can increase the resistance of mixtures to rutting and fatigue damage.

In recent years, increasing attention has been paid to the use of mineral wool waste as an additive to asphalt mixtures. Interest in this material stems not only from its reinforcing properties but also from the need to manage the growing amount of waste generated during the production, modernization, and demolition of buildings. Milat et al. [28] indicate that mineral wool waste is still recycled to a limited extent, with most of this type of waste ending up in landfills. Therefore, seeking new uses for this material is an important element in achieving sustainable construction goals. Research on the use of mineral wool fibers in asphalt mixtures indicates their beneficial effect on the performance properties of mixtures. The authors of [29] demonstrated improved moisture resistance, increased TSR, and improved Marshall parameters for mixtures containing rock wool fibers. Similar observations were presented by Zeng et al. [30], who found that waste rock wool creates a three-dimensional reinforcing structure in the mixture, improving resistance to water, permanent deformation, and high temperatures. At the same time, the authors point out that the effectiveness of fibers depends on their type, geometry and content in the mixture, and an excessive amount of the additive may lead to fiber agglomeration and deterioration of the structural homogeneity of MMAs [31].

Despite the growing number of publications, significant research gaps remain regarding the use of mineral wool in asphalt mixtures. Previous studies have focused primarily on glass fibers, basalt fibers, and waste rock wool from industrial processes or demolition materials. The potential use of loose granulated mineral wool commonly applied in blown-in insulation systems has received considerably less attention. There is also limited research on the impact of this type of additive on the properties of mixtures designed in accordance with national technical and material requirements for road wearing courses.

Therefore, the objective of this study was to evaluate the suitability of loose granulated mineral wool as a modifier of asphalt mixtures designed for wearing courses, with particular emphasis on its influence on mechanical and functional performance and compliance with current technical requirements.

## 2. Materials and Methods

### 2.1. Materials

**Figure 1:**
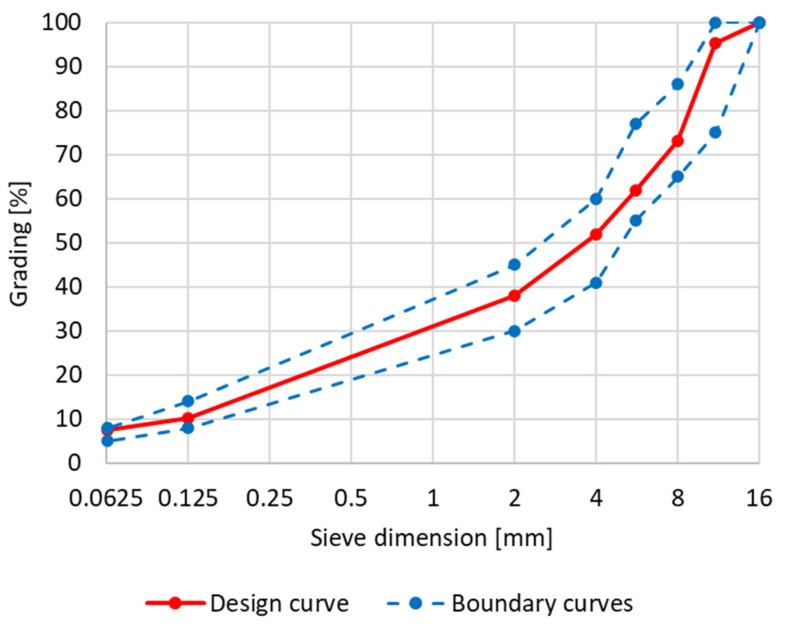
Grading curve.

To evaluate the influence of mineral wool on the properties of a mineral–asphalt mixture, an AC 11 S (AC—asphalt concrete with a maximum grain size of 11 mm, S—used for the wearing course) was used. The mixture was designed according to current technical requirements. The mix design incorporated basalt aggregate in fractions 0/2, 2/5, 5/8, and 8/11 mm, along with limestone filler. The proportions of individual aggregate fractions are presented in Table 1, and the grading curve is shown in Figure 1. Road asphalt 50/70 was used as the binder, from Orlen Asfalt, Poland. The asphalt parameters are presented in Table 2.

**Figure 2:**
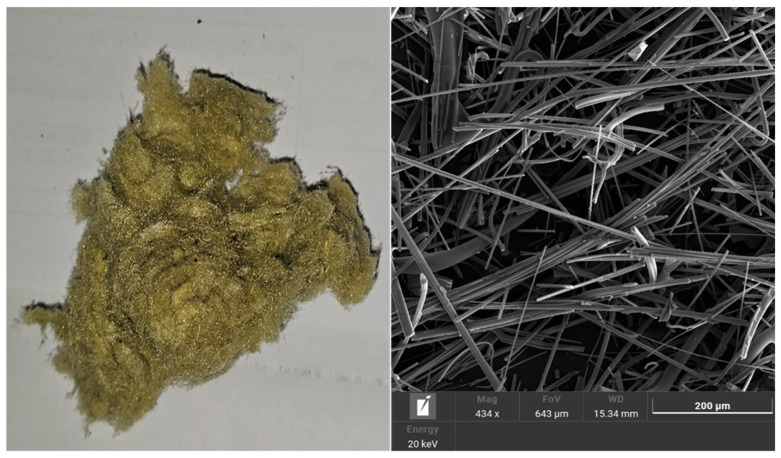
Granulated mineral wool.

Granulated basalt mineral wool from ROCKWOOL, Poland (bulk density: 55 kg/m^3^, high fire resistance), shown in Figure 2, was used in the study. Mineral wool is characterized by a fiber length ranging from 200 μm to 350 μm and a diameter of approximately 4 μm.

### 2.2. Research Methods

The scope of laboratory tests was selected to assess the suitability of mineral–asphalt mixtures modified with mineral wool for road pavement construction. For this purpose, the research included, in accordance with [32], air void content testing, Marshall stability testing, water and frost resistance testing, stiffness modulus testing, and resistance to permanent deformation testing.

**Figure 3:**
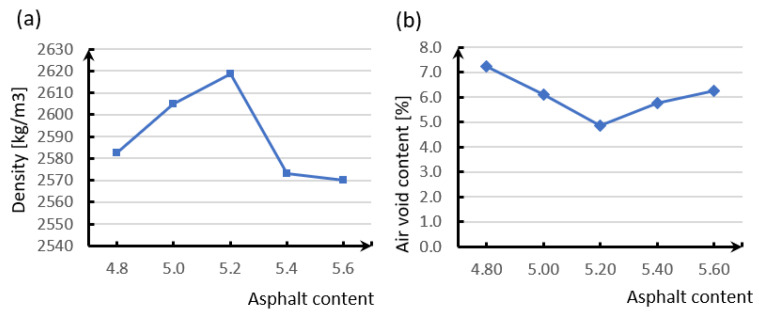
Optimum asphalt content, (**a**) bulk density results, (**b**) air void content results.

The optimal asphalt content was determined using the Marshall method. Laboratory samples included asphalt binder contents of 4.8%, 5.0%, 5.2%, 5.4%, and 5.6% in relation to the mineral–asphalt mixture. Based on the evaluation of bulk density and air void content, the optimal asphalt content was established at 5.2%. The dependence of density and air void content on the amount of asphalt content in the mineral-asphalt mixture is shown in Figure 3.

In the main tests, mineral wool was used as a modifier at 0.5%, 1.0%, and 1.2% by total mass of the MMA. Cylindrical specimens (100 × 63 mm) and slabs (305 × 305 × 40 mm) were prepared for testing. Sample preparation and compaction followed applicable standards depending on the test performed [32]. Prior to compaction, aggregate was conditioned at 160 °C for 8 h, and asphalt binder at 160 °C for 5 h in accordance with the requirements specified in EN 12697-35 [33]. After the required conditioning period for the aggregate and asphalt binder, the required amount of each aggregate fraction was weighed and placed in the mixing bowl. After pre-mixing the aggregate for 1 min, the required amount of mineral wool was added to the mineral mix and mixed again with the aggregate. Mixing time was also 1 min. After pre-mixing the mineral wool with the aggregate, the required amount of asphalt binder was added to the mix and mixed again until the aggregate was completely coated with the binder. The enveloping temperature was between 155 °C and 160 °C. After enveloping the asphalt mixture, test samples were prepared and compacted by tamping and rolling, depending on the test method, so that the compaction temperature was 150 °C.

Testing of the basic characteristics of the mixtures began with determination of air void content and bulk density of the samples in accordance with EN 12697-8 [34]. Cylindrical samples were compacted with 50 blows per side per EN 13108-20 [35]. Five replicates were prepared for each mineral wool content plus control samples (no wool).

The next test was the Marshall stability and flow test determined per EN 12697-34 [36] using cylindrical samples (Ø 101.5 × ~63 mm) compacted with 75 blows per side. Due to height deviations during compaction, a correction factor was applied:

(1) $$c=5.2e^{-0.0258\cdot h}$$

where

c—correction factor;

h—sample height [mm].

The next test was to determine the water and frost resistance of the mineral–asphalt mixture (ITSR) according to the procedure specified in EN 12697-12 [37]. For this test, the samples were compacted by ramming with 35 blows on each side. Six samples were prepared for each mineral wool content, along with control samples. Half of the samples, designated as the wet set, were conditioned according to the procedure specified in the standard, while the other half, designated as the dry set, were conditioned under room conditions. Water sensitivity was determined based on the following relationship:

(2) $$ITSR=100\cdot \frac{{ITS}_{w}}{{ITS}_{d}},$$

where

ITSR—indirect tensile strength index of the sample [%];

${ITS}_{w}$—average strength determined for the group of wet samples, rounded to the nearest integer [kPa];

${ITS}_{d}$—average strength determined for the group of dry samples, rounded to the nearest integer [kPa];

Elastic modulus (ITCY) was determined per EN 12697-24 [38] at 5 °C, 10 °C, 15 °C using cylindrical samples (Ø 101.5 × ~60 mm, 35 blows/side). Five replicates were tested per temperature and mineral wool content.

The final test performed was the resistance to permanent deformation test in accordance with EN 12697-22 [39]. The test was conducted according to procedure B in air at 60 °C. For the test, plate samples measuring 305 mm × 305 mm × 40 mm were prepared by compaction in a roller compactor according to compaction procedure C.1.20 specified in EN 13108-20. Two plate samples were prepared for the mineral wool content and control samples. For each sample, the average maximum rut RD_AIR_ was determined according to relationship (3) and the slope of the rutting curve WTS_AIR_ was determined according to relationship (4).

(3) $${RD}_{AIR}=\frac{\sum_{i=1}^{2}d_{10,000,i}}{2},$$

where

${RD}_{AIR}$—average maximum rut depth after 10,000 load cycles [mm],

${\mathrm{d}}_{10,000,i}$—rut depth after 10,000 load cycles and sample [mm],

(4) $${WTS}_{AIR}=\frac{d_{10,000}-d_{5000}}{5},$$

where

${WTS}_{AIR}$—rutting slope [mm/10^3^ cykli obciążeń],

$d_{10,000}$—rut depth after 10,000 load cycles [mm],

$d_{5000}$—rut depth after 5000 load cycles [mm].

## 3. Results

### 3.1. Air Void Content

**Figure 4:**
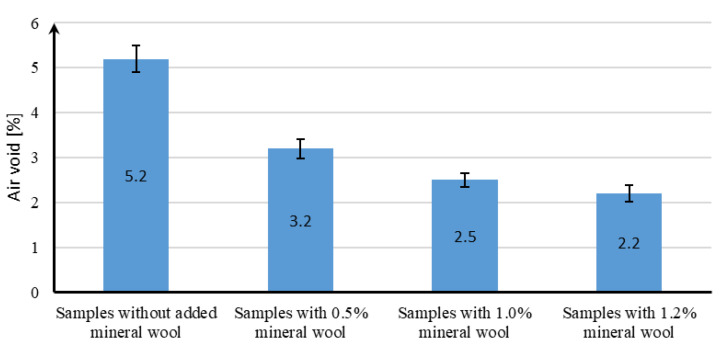
Air void content results.

**Figure 5:**
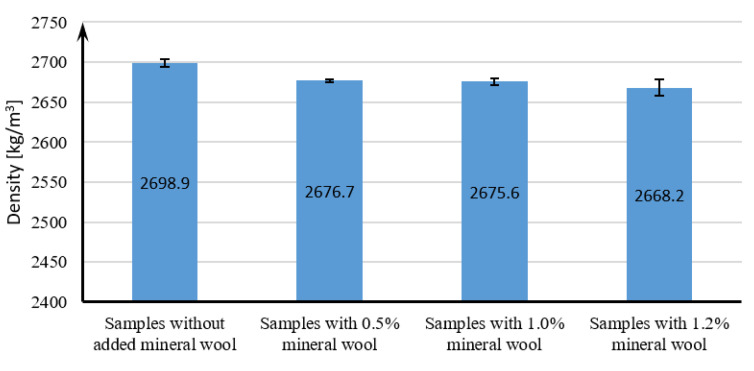
Bulk density test results.

The results of air void content and bulk density tests for mineral–asphalt mixtures with mineral wool addition are presented in Figure 4 and Figure 5.

Analysis of the results indicates that mineral wool addition significantly reduces air void content in mineral–asphalt mixtures (MMAs). As shown, air void content decreases with increasing wool dosage. A 0.5% addition reduced air voids by 38%, 1.0% by 52%, and 1.2% achieved the maximum 58% reduction compared to control samples. Notably, all modified mixtures met national requirements for asphalt concrete wearing courses (traffic categories KR3-4), where air void content must range from 2% to 4%.

Analysis of the results reveals that mineral wool addition reduces bulk density, although less significantly than air void content. Compared to control samples, 0.5% wool decreased bulk density by 22.2 kg/m^3^ (0.8%), 1.0% wool by 23.3 kg/m^3^ (0.9%), and 1.2% wool achieved the maximum reduction of 30.7 kg/m^3^ (1.1%). These minimal density losses (≤1.1%) confirm that mineral wool does not adversely affect the bulk density of MMAs.

### 3.2. Marshall Stability and Flow

Marshall stability and flow tests were performed after conditioning the samples in water at 60 °C for 30 min. The test results are presented in Table 3.

Analysis of the results shows that mineral wool negatively affects both Marshall stability and flow compared with the reference mixture. The use of 0.5% mineral wool reduced stability by 11%, while 1.0% mineral wool reduced stability by 13%. It is noteworthy that at the highest mineral wool content of 1.2%, the decrease in stability was only 4%. The situation is quite different for flow. In this case, 0.5% mineral wool increased flow by 127%, while 1.2% mineral wool increased flow by 118%. The least negative effect on flow was observed for 1.0% mineral wool, which increased flow by 55%.

As the results show, mineral wool significantly worsens the Marshall test results for asphalt concrete samples. However, because the technical documents for asphalt mixtures used in national roads do not specify requirements for stability and flow, the use of mineral wool in asphalt concrete cannot be ruled out based on this single test alone.

### 3.3. Water and Frost Resistance

**Figure 6:**
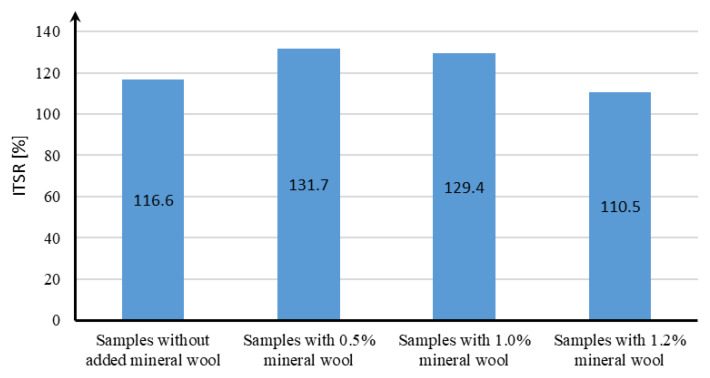
Water and frost resistance.

Water and frost resistance was determined using the Indirect Tensile Strength Ratio (ITSR), comparing dry-conditioned and wet-conditioned samples. Results as a function of mineral wool content are presented in Figure 6.

Analysis of the presented results indicates that water and frost resistance decreases with increasing mineral wool content. The presented data show that samples with 0.5% and 1.0% mineral wool exhibited water and frost resistance greater than reference samples by 15.1% and 12.8%, respectively. In contrast, samples with 1.2% mineral wool content resulted in a 6.1% decrease in water and frost resistance. It is noteworthy that both reference samples and samples with mineral wool addition showed higher indirect tensile strength for the wet set (samples exposed to water and sub-zero temperatures), as evidenced by the ITSR index exceeding 100%. Therefore, in each case, there was an increase in strength rather than a decrease. Only for samples with the highest mineral wool content was this increase the smallest, reaching 110.5%. The greatest increase in wet sample strength compared to dry samples was observed for 0.5% and 1.0% wool content, amounting to 131.7% and 129.4%, respectively. For reference samples, the resistance increase was 116.6%.

**Figure 7:**
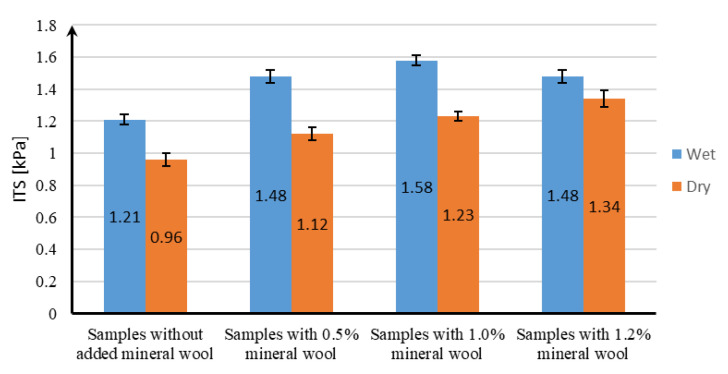
Water and frost resistance—dry vs. wet samples.

To illustrate the actual differences in the obtained results, Figure 7 presents indirect tensile strength results divided into dry and wet samples.

Analysis of the presented data indicates that the indirect tensile strength of dry samples increases with increasing mineral wool content. Samples with 0.5% wool exhibited 17% higher strength than samples without wool, while 1.0% wool samples showed 28% higher strength. The largest strength increase of 40% compared to reference samples was observed for 1.2% wool samples. For wet-conditioned samples (water exposure), greater strength was also observed with mineral wool addition compared to samples without wool. In this case, 1.0% wool samples showed the largest strength increase of 31% compared to reference samples. The 0.5% and 1.2% wool samples exhibited strength increases of 22%.

### 3.4. Stiffness Modulus Test

**Figure 8:**
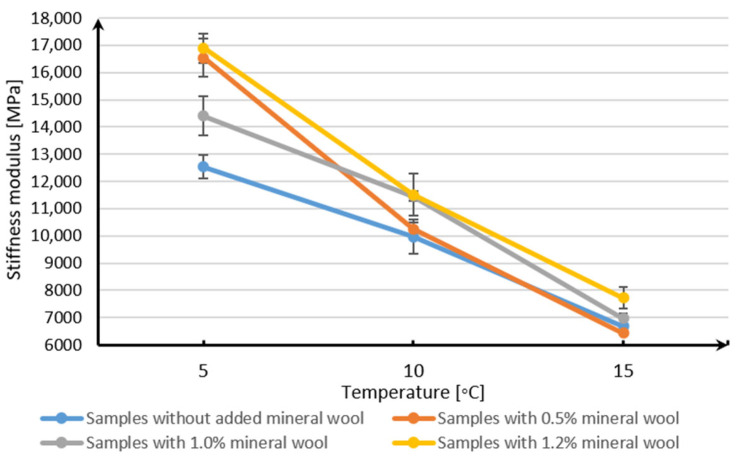
Stiffness modulus results.

The stiffness modulus was determined for all samples at control temperatures of 5 °C, 10 °C, and 15 °C. These temperatures were selected to characterize the behavior of mineral wool-reinforced asphalt concrete during spring and summer periods. Additionally, the influence of mineral wool on stiffness modulus at lower temperatures—where asphalt concrete stiffening may occur—was assessed. Results are presented in Figure 8.

Analysis of the presented data indicates that at lower temperatures, mineral wool addition has a greater effect on stiffness modulus than at higher temperatures. At the 5 °C reference temperature, all samples with mineral wool exhibited significantly higher stiffness moduli than reference samples. The largest increases—32% (0.5% wool) and 35% (1.2% wool)—were observed for those contents. Samples with 1.0% wool showed a 15% increase (half that of the others). At 10 °C, the largest 15% increase occurred for 1.0% and 1.2% wool samples, while 0.5% wool showed only a 3% increase. At 15 °C, only 1.2% wool samples achieved a 15% increase; 1.0% wool showed a 4% increase, while 0.5% wool exhibited a 4% decrease compared to reference samples. The results demonstrate that 1.2% mineral wool content provides the most pronounced and stable stiffness modulus increase. Mineral wool positively affects asphalt concrete stiffness modulus; however, larger increases were expected at higher temperatures. Contrary to expectations, greater increases occurred at lower temperatures and smaller ones at higher temperatures.

### 3.5. Permanent Deformation Resistance

**Figure 9:**
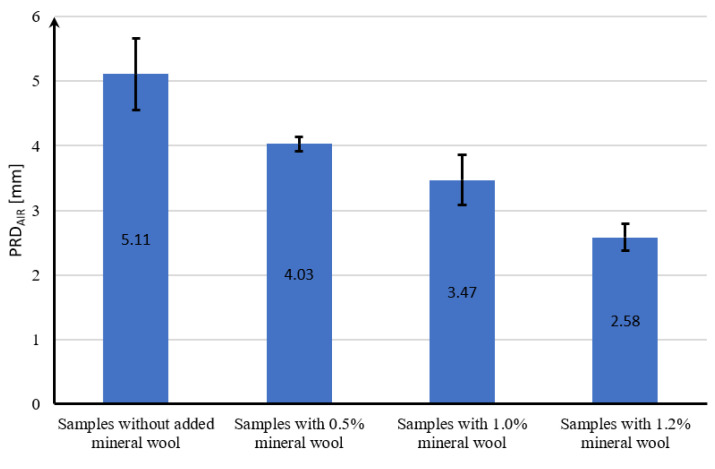
Maximum rut depth results.

**Figure 10:**
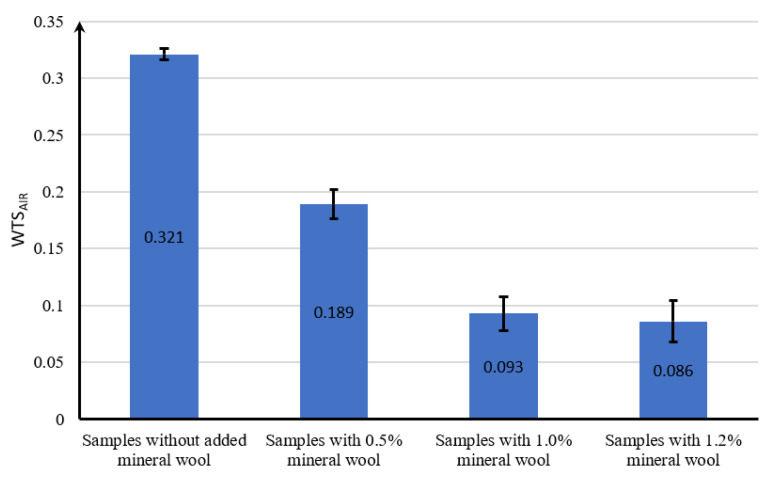
Rutting slope results.

Permanent deformation resistance was assessed via wheel-tracking tests on slab samples compacted using roller compactors simulating field compaction during placement. Maximum rut depth results are shown in Figure 9, and rutting slope results in Figure 10.

Analysis of the results clearly shows that maximum rut depth decreases with increasing mineral wool content. For 0.5% wool samples, maximum rut depth decreased by 21% compared to reference samples. The 1.0% wool samples showed a 32% reduction, while samples with the highest wool content (1.2%) achieved the largest 50% reduction. These results unequivocally demonstrate that mineral wool addition positively affects resistance to permanent deformation.

Analysis of rutting slope results confirms that mineral wool addition positively affects performance by reducing the slope—indicating smaller rut progression between 5000 and 10,000 load cycles. For 0.5% wool samples, rutting slope decreased by 41% compared to reference samples. The 1.0% and 1.2% wool samples showed very similar reductions of 71% and 73%, respectively—a highly positive effect.

Mineral wool has a much greater impact on rutting slope reduction than on maximum rut depth, directly contributing to favorable properties for mineral–asphalt mixtures intended for road pavements.

## 4. Discussion

The test results unequivocally confirm that mineral wool addition beneficially affects mineral–asphalt mixture (MMA) properties. Mixtures containing mineral wool exhibited significantly better water and frost resistance than reference mixtures. The increased ITSR values indicate improved binder–aggregate adhesion and reduced structural degradation under moisture and freeze–thaw cycles—crucial for pavement durability in Polish climatic conditions. It should also be noted that a similar increase in water and frost resistance was observed by the authors of the study [30] with mineral wool contents of 0.2% and 0.3%. This translates directly into the benefits of using mineral wool in mineral–asphalt mixtures and at the same time determines the need to conduct experimental tests for locally available materials, which differ significantly.

Stiffness modulus analysis revealed that mineral wool significantly increases mixture stiffness across the tested temperature range (5 °C, 10 °C, 15 °C). Enhanced elastic stiffness demonstrates improved dynamic load transfer from vehicle traffic, directly contributing to better fatigue resistance. Importantly, results show no excessive stiffening that could increase low-temperature cracking susceptibility. The beneficial effect of rock wool on the stiffness of the mixture was also presented in [20], where a 0.3% wool content resulted in a 21% increase in stiffness. When the wool content increased to 0.5%, the authors observed a slight decrease in stiffness compared to the maximum value, but the mixtures containing mineral wool were still characterized by a stiffness increased by approximately 17%. Similar increases in stiffness were also observed for the addition of glass fibers [26].

Significant improvement was also observed in permanent deformation resistance. Wheel-tracking tests demonstrated that 1.2% mineral wool achieved approximately 50% lower maximum rut depth compared to reference mixtures. This indicates effective reduction in permanent deformation susceptibility, particularly valuable under heavy traffic and high pavement temperatures. Similar properties were found by the authors of the work [29], in which the use of mineral wool in the amount of 0.8% showed the most favorable effect on the resistance to permanent deformation of mineral–asphalt mixtures.

Notably, mineral wool addition did not negatively affect bulk density, enabling mechanical property enhancement without increasing asphalt binder content. This offers economic and technological advantages by reducing production costs while maintaining consistent operational performance.

Mineral wool proves an effective modifier for improving road pavement durability through enhanced environmental resistance, reduced fatigue degradation, improved rutting resistance, and mix structure stabilization.

Implementation considerations include precise dosing and uniform distribution for repeatable results, plus adjusted mixing and compaction parameters due to fiber effects on production technology.

Conclusions: Mineral wool is a viable additive for wearing course asphalt mixtures, offering increased durability, lower maintenance costs, and improved user safety. Results justify further long-term performance studies and economic feasibility assessments for full-scale road applications. Determining the optimal mineral wool content in asphalt mixes remains crucial. In most literature studies, mineral wool was added at up to 0.8% of the asphalt mix. It should be noted that even a 2% addition of mineral wool had a positive effect on the strength test results [32]. Analyzing the literature studies and those conducted by the authors, it should be noted that determining the mineral wool content depends directly on the expected benefits and characteristics of the mix. The optimal mineral wool content cannot be clearly determined. Different content will be beneficial when optimizing the mix for the energy required for installation, different for the durability of the surface during use, and still different for the changing climatic conditions affecting the surface. It should be noted that the maximum aggregate size used and the asphalt content in the mix will have a direct impact on the mineral wool content.

## 5. Conclusions

Based on the laboratory tests carried out to assess the suitability of granulated mineral wool as a modifier of the mineral–asphalt mixture for the wearing course, the following conclusions can be drawn:

1. The conducted research confirmed that granulated mineral wool can be effectively used as a modifying additive in mineral–asphalt mixtures, contributing to the improvement of their functional and strength properties, thus achieving the assumed research goal.
2. The use of mineral wool improves the mix’s structural integrity without having to change its mineral composition or asphalt binder content. Reducing void content increases the surface’s durability and reduces its susceptibility to weathering.
3. The addition of mineral wool significantly increases the resistance of mixtures to water and frost, which indicates an improvement in the durability of the asphalt–aggregate bond and a reduction in degradation processes occurring during the use of the surface in climatic conditions typical of Poland.
4. The increased stiffness modulus and improved resistance to permanent deformation confirm that the use of mineral wool increases the mix’s ability to withstand road traffic loads. This translates into a longer service life and reduced maintenance frequency.
5. Despite the observed decrease in Marshall stability and increase in settlement values, these changes did not limit the additive’s beneficial effect on the remaining analyzed properties. This indicates that this parameter is not a decisive factor in assessing the suitability of mineral wool as a modifying additive.
6. The most beneficial effects in terms of improving mechanical and operational properties were achieved with a mineral wool addition of approximately 1.2% by weight of the asphalt mixture. However, the results indicate that the optimal additive content should be determined on a case-by-case basis, taking into account the mix type, the properties of the materials used, and the expected operational parameters.
7. The research results indicate that the use of granulated mineral wool can be an effective solution supporting the design of more durable road surfaces, characterized by greater resistance to environmental factors and operational deformations.
8. Further research should focus on assessing the long-term performance of mixtures with mineral wool in real operating conditions and on determining the relationship between the optimal content of the additive and the composition of the mineral–asphalt mixture.

## Figures and Tables

**Table 1:** Aggregate composition.

Aggregate	Content [%]
Limestone filler	5%
Basalt 0/2	40%
Basalt 2/5	15%
Basalt 5/8	10%
Basalt 8/11	30%

**Table 2:** Properties of 50/70 asphalt.

Property	Unit	Value
Penetration at 25 °C	0.1 mm	50–70
Softening point	°C	46–54
Aging resistance at 163 °C		-
Retained penetration	%	≥50
Increase in softening point	°C	≤9
Mass change (absolute value)	%	≤0.5
Flash point	°C	≥240
Solubility	% (m/m)	≥99.0

**Table 3:** Stability and settlement test results.

Wool Content	Stability/Standard Deviation [kN]	Flow/ Standard Deviation [mm]
Reference samples	8.2/0.21	3.3/0.19
Samples with 0.5% wool	7.3/0.15	7.5/0.38
Samples with 1.0% wool	7.1/0.17	5.1/0.12
Samples with 1.2% wool	7.9/0.10	7.2/0.57

## Data Availability

The original contributions presented in this study are included in the article. Further inquiries can be directed to the corresponding author.
